# New Insights into the Role of KLF10 in Tissue Fibrosis

**DOI:** 10.3390/ijms25021276

**Published:** 2024-01-20

**Authors:** Uzma Yaseen, Soonjae Hwang, Sangbin Park, Soo-Bin Kim, Ho-Jae Lee, Ji-Young Cha

**Affiliations:** 1 Department of Health Sciences and Technology, GAIHST, Gachon University, Incheon 21999, Republic of Korea; uzmayaseen255@gmail.com (U.Y.); tree5267@gachon.ac.kr (S.P.); orot0818@naver.com (S.-B.K.); 2 Department of Biochemistry, Lee Gil Ya Cancer and Diabetes Institute, College of Medicine, Gachon University, Incheon 21999, Republic of Korea; soonjae@gachon.ac.kr

**Keywords:** fibrosis, Krüppel-like factor 10, TGF-β, NAFLD, NASH

## Abstract

Fibrosis, characterized by excessive extracellular matrix accumulation, disrupts normal tissue architecture, causes organ dysfunction, and contributes to numerous chronic diseases. This review focuses on Krüppel-like factor 10 (KLF10), a transcription factor significantly induced by transforming growth factor-β (TGF-β), and its role in fibrosis pathogenesis and progression across various tissues. KLF10, initially identified as TGF-β-inducible early gene-1 (TIEG1), is involved in key biological processes including cell proliferation, differentiation, apoptosis, and immune responses. Our analysis investigated KLF10 gene and protein structures, interaction partners, and context-dependent functions in fibrotic diseases. This review highlights recent findings that underscore KLF10 interaction with pivotal signaling pathways, such as TGF-β, and the modulation of gene expression in fibrotic tissues. We examined the dual role of KLF10 in promoting and inhibiting fibrosis depending on tissue type and fibrotic context. This review also discusses the therapeutic potential of targeting KLF10 in fibrotic diseases, based on its regulatory role in key pathogenic mechanisms. By consolidating current research, this review aims to enhance the understanding of the multifaceted role of KLF10 in fibrosis and stimulate further research into its potential as a therapeutic target in combating fibrotic diseases.

## 1. Introduction

Fibrosis is a critical pathological process characterized by the aberrant accumulation of extracellular matrix (ECM) components in response to chronic tissue injury or persistent inflammation [1,2,3,4,5,6]. It starts as a protective mechanism aimed at wound repair and restoration of tissue integrity but can evolve into a detrimental condition leading to significant scarring, loss of organ function, and eventual organ failure [1,2,3,4,5,6]. This complex process is a hallmark of numerous chronic diseases and can progress to liver failure, heart dysfunction, kidney failure, and severe respiratory dysfunction depending on the affected organ. Fibrosis is also a major pathological feature of many chronic autoimmune diseases, including rheumatoid arthritis, systemic scleroderma, systemic lupus erythematosus, Crohn’s disease, ulcerative colitis, and myelofibrosis. The widespread prevalence and severe impact of these diseases highlight the urgent need for a deep understanding of their underlying molecular mechanisms and potential therapeutic targets.

**Figure 1:**
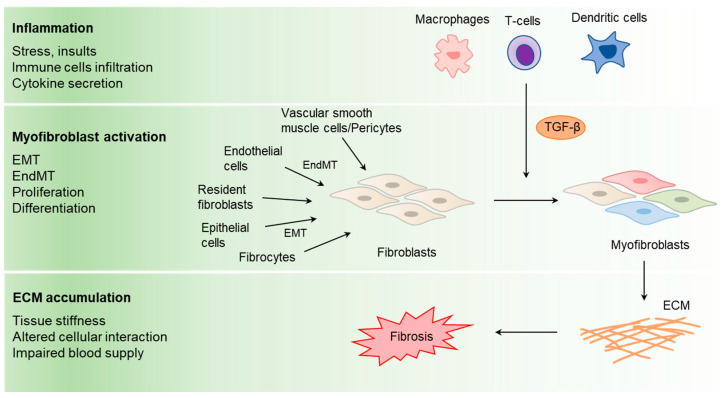
The pathological process of fibrosis. Fibrosis commences with an inflammatory response triggered by various stressors, insults, and the infiltration of immune cells, which in turn secrete cytokines. A pivotal event in this sequence is the activation of myofibroblasts which arises through multiple mechanisms, including EndMT, EMT, and the proliferation and differentiation of resident fibroblasts and endothelial cells. Myofibroblasts can originate from various cellular sources, with vascular smooth muscle cells/pericytes undergoing EndMT and epithelial cells transitioning through EMT to fibrocytes before differentiating into myofibroblasts. This transition is further influenced by immune cells such as macrophages, T-cells, and dendritic cells, which secrete TGF-β, a key cytokine that facilitates the transformation into myofibroblasts. Subsequently, these myofibroblasts are instrumental in the accumulation of the ECM, leading to increased tissue stiffness, disrupted cellular interactions, and impaired blood supply. The pathological endpoint of these interconnected processes is fibrosis, characterized by the excessive and disruptive deposition of ECM, ultimately impairing the normal architecture and function of the tissue.

Although the progression and impact of fibrosis varies with each organ system, the underlying pathological basis remains consistent, characterized by chronic inflammation, activation of fibroblasts, excessive deposition of ECM, and dysregulated remodeling and repair processes (Figure 1) [2,3,4,6]. The initiation of fibrosis is often triggered by various factors such as infection, autoimmune reactions, mechanical stress, or toxic insults [1]. These events set off a cellular cascade aimed at repairing the damaged tissue. The acute inflammatory response that follows brings immune cells like macrophages and lymphocytes to the injury site [6]. These cells are not only essential in the initial defense and clearance of debris but also secrete cytokines and growth factors, most notably transforming growth factor-β (TGF-β), platelet-derived growth factor (PDGF), and connective tissue growth factor (CTGF), that coordinate the fibrogenic response [7,8,9].

Central to fibrosis is the activation of fibroblasts, which are prevalent in most tissues. Under the influence of cytokines, particularly TGF-β, these fibroblasts transform into myofibroblasts (Figure 1) [6]. Fibroblasts involved in fibrosis can originate from vascular smooth muscle cells, pericytes, fibrocytes, endothelial and epithelial cells, or resident fibroblasts, while emerging myofibroblasts/activated fibroblasts excessively synthesize and secrete ECM proteins that contribute to fibrosis [10]. Depending on their origin, these cells undergo proliferation, differentiation, and endothelial/epithelial-to-mesenchymal transition (EndMT/EMT). Myofibroblasts, marked by their expression of α-smooth muscle actin (α-SMA), become prolific producers of ECM components, especially collagen. They are more contractile than regular fibroblasts, contributing to the increased stiffness and altered mechanical properties of the affected tissue [7,11,12,13,14]. This excessive ECM deposition is exacerbated by an imbalance in matrix metalloproteinases (MMPs) and tissue inhibitors of metalloproteinases (TIMPs) [15,16]. Reduced tissue elasticity, altered cellular interactions, and impaired blood supply are clinical consequences of fibrosis, making it a significant clinical concern.

In recent years, Krüppel-like factor 10 (KLF10), initially designated as TGF-β-inducible early gene-1 (TIEG1), has emerged as a critical player in fibrosis [17,18,19]. As a member of the KLF family of zinc-finger transcription factors, KLF10 is significantly induced by TGF-β and is implicated in various biological processes such as cell proliferation, differentiation, apoptosis, and immune responses [20]. The role of KLF10 in fibrosis has gained attention, particularly its interaction with pivotal signaling pathways, including TGF-β/SMAD3, ER stress, and metabolic reprogramming [18,21]. Although the precise mechanisms and downstream targets of KLF10 in fibrosis are still being unraveled, its potential as a therapeutic target against fibrotic diseases is becoming increasingly recognized.

This review aims to thoroughly examine the role of KLF10 in tissue fibrosis. By analyzing recent studies, we seek to provide a deep understanding of KLF10 characteristics, including its gene and protein structures, as well as its interaction partners. Moreover, we examine its context-dependent functions in various fibrosis-affected tissues and organs. By integrating the existing knowledge, we hope to identify the potential mechanisms through which KLF10 regulates fibrotic processes and discuss its therapeutic implications.

## 2. KLF10 Characteristics

KLFs, named after the *Drosophila melanogaster* protein Krüppel, are a family of Sp1-like transcription factors characterized by zinc-finger domains that regulate various cellular processes, including proliferation, development, and apoptosis. The KLF family currently comprises 18 members with diverse expression profiles across multiple tissues [22,23]. KLF10, initially identified as an early gene induced after TGF-β treatment in human fetal osteoblast cells (hFOB), was named TIEG1 [17]. Thus, its functions can be investigated in various biological contexts. Notably, KLF10 is expressed in several tissues, including the liver, pancreas, adipose tissue, and skeletal muscles [24].

### 2.1. KLF10 Gene Structure and Variations

**Figure 2:**
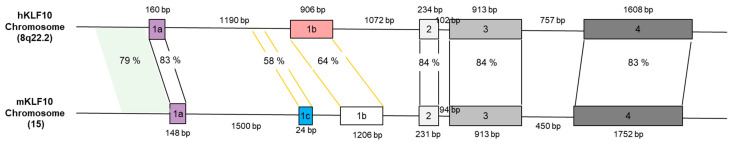
*KLF10* gene structure of mice and humans. Exon sequence similarity is represented by black lines. The light green box indicates 79% sequence conservation between the mouse and human *KLF10* genes over 500 bp upstream of the transcription start sites. The orange lines depict identical sequences in mouse exon 1c and human *KLF10* gene (58%), as well as human exon 1b and mouse *klf10* gene (64%). Exons are represented by rectangles with base pair length. In the mouse gene, the blue rectangle signifies a sequence obtained from an expressed sequence tag. An open white rectangle represents a region of the mouse gene that might encode *mEGRα*. The transcript information has been obtained using Ensembl database [27].

The *KLF10* gene, localized on the 8q22.2 locus of the human chromosome, spans approximately 7.0 kb and comprises four exons and their corresponding introns [25]. In other species such as *Mus musculus* and *Rattus norvegicus*, *klf10* is located on chromosomes 15 and 7q22, respectively [26]. A comparison of the human and mouse *KLF10* genes revealed significant sequence similarity in the exons (Figure 2). The mouse gene, which extends over approximately 6.3 kb, contains four exons, with exon 1a being 9 bp shorter than its human counterpart. Additionally, the mouse gene contains an extra exon, 1c. Zinc-finger motifs crucial for DNA binding are located in exons 3 and 4.

**Figure 3:**
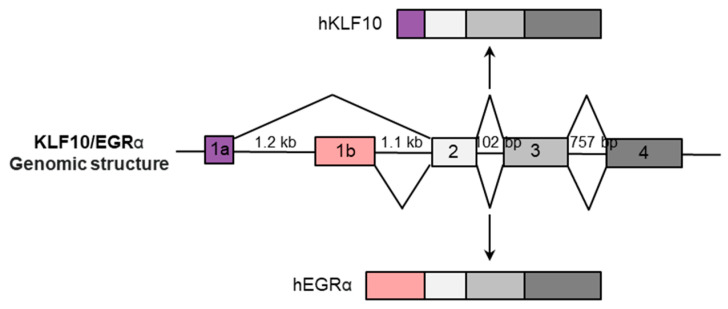
*KLF10/EGRα* gene structure. The *KLF10/EGRα* gene is 8 kb long and has four exons (represented by colorful rectangles). Alternative first exons result in *hKLF10*-specific 1a (**top**) or *hEGRα*-specific 1b (**bottom**). Introns 1, 2, 3, and 4 are 1.6 kb, 1.1 kb, 103 bp, and 758 bp long, respectively.

The *hKLF10* splice variant, termed early growth response α (*hEGRα*), was identified in human prostate cancer cells [28]. Both *hKLF10* and *hEGRα* originate from the same gene but use distinct promoters, resulting in proteins differing by only 12 amino acids at their N-termini (Figure 3). The primary sequence similarity, except for the first exon, is noteworthy: exon 1a (159 base pairs) aligns with *KLF10*, while exon 1b (471 base pairs) is specific to *EGRα*. Mouse *klf10* lacks an *EGRα*-specific exon but includes the unique exon 1c, suggesting the possibility of an unidentified mouse transcript [28,29].

Computational analysis of the 5′-upstream regions of *KLF10* and *EGRα* reveals the absence of a canonical TATA box but identifies a consensus sequence (5′-GGTGTG-3′) and binding sites for transcription factors such as JunB, c-myc, and Sp1. For instance, the *KLF10* gene promoter contains a JunB binding site, which facilitates KLF10 transcription [30].

### 2.2. KLF10 Protein Structure

**Figure 4:**
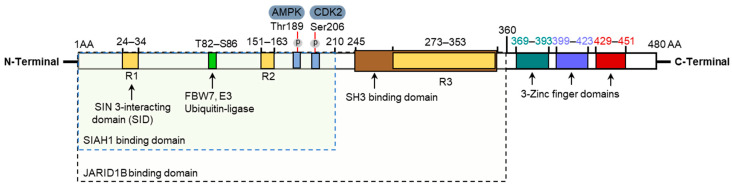
hKLF10 protein structure with different interaction partners and phosphorylation sites. hKLF10 protein has 480 amino acids with three zinc-finger domains at the C-terminal, three repression (R1, R2, and R3) domains, and an SH3-binding domain at the N-terminal. It has binding domains for Seven in Absentia homologue-1 (SIAH1; 1–210), a Jumonji AT-rich domain 1B/lysine-specific demethylase 5 B (JARID1B; 1–360), and AMP-activated protein kinase (AMPK) and cyclin-dependent kinase 2 (CDK2) phosphorylation sites at Thr189 and Ser206, respectively.

KLF10 is a 480 amino acid protein with an approximate molecular weight of 72 kDa. It is rich in proline and serine residues, constituting 12.8% and 11.6% of its total amino acid composition, respectively [31]. The protein structure includes several functional domains (Figure 4). The N-terminus contains R1 (10 amino acids), R2 (12 amino acids), and R3 (approximately 80 amino acids) repression domains and a proline-rich sequence [32]. The R1 domain is crucial for interacting with the co-repressor mSin3A, inhibiting the transcriptional activation of target genes through histone deacetylation and subsequent chromatin remodeling [33]. Therefore, the R1 domain is commonly referred to as the mSin3A interacting domain. Additionally, KLF10 possesses multiple proline-rich Src homology-3 (SH3)-binding domains for interaction with proteins such as Sp1, facilitating transcriptional activation [34]. The C-terminus features three conserved C2H2 zinc-finger motifs crucial for DNA binding, separated by a seven-amino-acid spacer region [31]. By binding to specific GC-rich Sp1-like cis-regulatory sequences, KLF10 regulates the transcriptional activity of target genes. Notably, the N-terminus of KLF10 is highly dynamic and distinct from that of other genes in the GenBank database, except KLF11, which contrasts with the shared zinc-finger motif of the C-terminus with Sp family transcription factors [17].

### 2.3. KLF10 Activation, Interactions, and Protein Stability

KLF10 is a versatile transcription factor whose activity and downstream effects are regulated through interaction with various proteins including Jumonji AT-rich domain 1B/lysine-specific demethylase 5 B (JARID1B/KDM5B), Seven in Absentia homologue-1 (SIAH1), FBW7, cyclin-dependent kinase 2 (CDK2), and AMP-activated protein kinase (AMPK) (Figure 4).

JARID1B/KDM5B was the first identified interaction partner of KLF10, interacting within amino acid 1–360 [35]. As a transcriptional repressor, KLF10 recruits JARID1B/KDM5B to the *SMAD7* promoter, removing methyl groups from trimethylated lysine 4 on histone H3, thereby inhibiting transcriptional initiation [35]. SMAD7 inhibits intracellular TGF-β signaling; thus, the interaction between KLF10 and JARID1B may enhance the tumor suppressive effects of TGF-β.

Johnsen et al. discovered that KLF10 protein stability is controlled via ubiquitination by its interaction partner SIAH1 [36]. SIAH1, a ubiquitin ligase, participates in TGF-β/SMAD signaling activation by binding to the amino acids 1–210 at the N-terminus of KLF10. SIAH1 co-expression inhibits TGF-β signaling by proteasomal degradation of KLF10, thus limiting TGF-β signaling magnitude and interval [36]. FBW7 is another E3 ubiquitin ligase involved in KLF10 protein degradation. FBW7 can bind to Thr82–Ser86 of KLF10 and mediate *SMAD7* expression [37]. CDK2 phosphorylates KLF10 at Ser206, disrupting its association with SIAH1 and preventing proteasomal degradation [38], thus linking cell cycle progression to increased KLF10 protein levels.

AMPK is a key metabolic regulator that phosphorylates KLF10 at Thr189, thereby modulating its stability and transcriptional activity [39]. These interactions with JARID1B/KDM5B, SIAH1, FBW7, CDK2, and AMPK demonstrate the complex regulatory network of KLF10 in TGF-β signaling and related pathways. The interplay between KLF10 and its interaction partners finely tunes gene expression and modulates various cellular processes. Further investigation is necessary to fully understand the functional significance of these interactions in tissue fibrosis and other biological contexts.

## 3. KLF10 Functional Regulation

KLF10, initially recognized as a key TGF-β signaling mediator, plays diverse roles in cell biology, notably in inhibiting cell proliferation, promoting apoptosis, and regulating energy metabolism. These functions suggest its role as a tumor suppressor and its involvement in coordinating metabolic processes with the circadian clock and in responding to nutrient availability.

### 3.1. TGF-β Regulation

**Figure 5:**
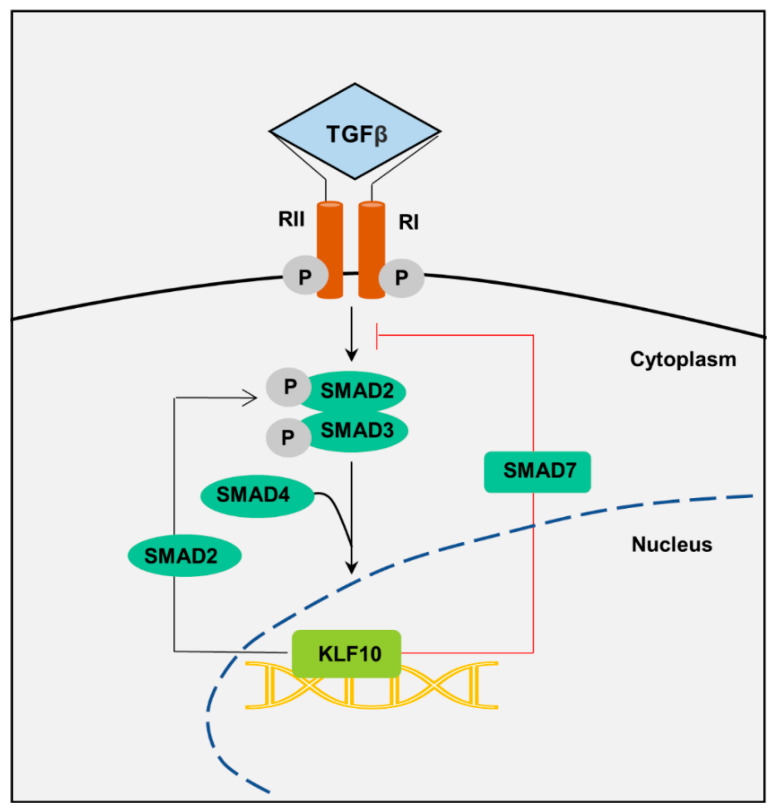
KLF10 as a TGF-β/SMAD signaling regulator. TGF-β binds to its receptors and triggers R-SMADs phosphorylation. This activated TGF-β/SMAD signaling enhances *KLF10* expression, which further activates TGF-β/SMAD signaling through transcriptional activation of *SMAD2* and repression of *SMAD7*.

KLF10 responds to TGF-β and related family members, such as activin A, bone morphogenetic protein (BMP) 2, BMP4, and glial cell-derived neurotrophic factor, making it a crucial participant in the canonical TGF-β signaling pathway [17,25,40]. The signaling cascade begins when TGF-β ligand binds to a heterodimeric receptor complex comprising TβRII and TβRI (Figure 5). TβRII activates TβRI through phosphorylation, catalyzing SMAD2/SMAD3 (R-SMADs) phosphorylation. This phosphorylated complex, along with SMAD4, translocates to the nucleus and stimulates *KLF10* expression. KLF10 then modulates this pathway, particularly by inhibiting *SMAD7* transcription, a key negative regulator of TGF-β signaling [41]. TGF-β also triggers physiological responses via non-canonical SMAD signaling pathways involving proteins such as mitogen-activated protein kinase (MAPK) and NF-κB [42].

KLF10 overexpression increases apoptosis and reduces the proliferation of various cell types, including human osteosarcoma cells [43,44,45,46]. This regulation is predominantly SMAD2-dependent, affecting its phosphorylation, and affects sparing SMAD3 and SMAD4 [47]. KLF10 influences the apoptotic pathway by upregulating Bax/Bim and downregulating Bcl-2/Bcl-XL, thus triggering mitochondrial disturbances and caspase 3 activation [43]. KLF10 also modulates *TβRII* expression in CD8+ T lymphocytes [48] and murine macrophages [49], demonstrating its broad regulatory impact across different cell types.

### 3.2. Nutrient and Metabolic Pathway Regulation

The liver, as a primary metabolic organ, operates under a complex network of regulatory mechanisms that are sensitive to nutritional states such as feeding, fasting, and dietary variations. Among these regulators, KLF10 plays a critical role in regulating metabolic pathways, particularly those influenced by circadian rhythms, carbohydrate response element-binding protein (ChREBP), and AMPK.

KLF10 is deeply intertwined with circadian rhythms, which are fundamental in orchestrating metabolic processes [50,51]. The oscillatory nature of the circadian clock regulates the expression of *KLF10*, aligning it with daily metabolic cycles [51]. KLF10 also exhibits robust circadian expression in the liver, regulated by core clock proteins such as BMAL1. This circadian regulation is crucial for maintaining metabolic homeostasis, emphasizing the importance of KLF10 in synchronizing liver metabolism with circadian rhythms [51]. Beyond circadian control, KLF10 is also responsive to nutrient signaling, particularly to fluctuations in glucose levels. Ruberto et al. demonstrated that glucose and fructose induce *Klf10* expression in the liver [52]. This induction plays a key role in mitigating metabolic challenges such as glucose intolerance and hepatic steatosis, especially under conditions of high sugar intake. Thus, KLF10 acts as a transcriptional moderator, integrating nutritional signals into the metabolic regulatory network [52].

ChREBP is a glucose-induced transcription factor that is stimulated by dietary carbohydrates. Fasting suppresses ChREBP activation, whereas refeeding with a high-carbohydrate diet stimulates ChREBP expression and activity [53,54]. ChREBP directly regulates KLF10 expression by binding to ChoRE in the *KLF10* promoter region [55,56]. KLF10 deletion increases the activity of ChREBP target genes in the liver, while KLF10 overexpression inhibits ChREBP target genes. The interaction between KLF10 and ChREBP, a central glucose metabolism regulator, forms a feedback loop that is crucial for maintaining hepatic metabolic homeostasis.

AMPK, a cellular energy sensor, integrates metabolic pathways in response to energy demand [57]. KLF10, functioning as a substrate for AMPK, plays a crucial role in the regulation of hepatic lipogenesis [39]. It acts as a post-translational repressor of SREBP1c and its associated genes involved in lipogenesis. When phosphorylated by AMPK, KLF10’s ability to repress *Srebp1* transcription is enhanced, leading to a reduction in lipogenesis in HepG2 cells [39]. This mechanism indicates that under low-energy conditions, AMPK activation leads to the phosphorylation of KLF10, which in turn suppresses energy-intensive metabolic processes. These findings reveal that KLF10 expression is not only regulated by dietary nutrients but also influenced by a network of factors including ChREBP, AMPK, and SREBP1, all of which contribute to the control of hepatic metabolism.

### 3.3. Inflammation and Metabolic Regulation

CD4+ T-cells, particularly regulatory T-cells (Tregs), are crucial for controlling inflammation and metabolic processes in obesity. An imbalance in Treg cells contributes to insulin resistance and diabetes. In CD4+-T-cell-specific KLF10 knockout mice, a predisposition to obesity, insulin resistance, and fatty liver was observed, attributed to impaired CD4+ Treg mobilization to liver and adipose tissues and reduced TGF-β3 release [58]. This deficiency in CD4+-T-cell-specific KLF10 knockout Tregs was linked to reduced mitochondrial respiration, glycolysis, and PI3K–Akt–mTOR signaling, thereby affecting their chemotactic abilities. This study underscores that CD4+ T-cell KLF10 is a key regulator of obesity and insulin resistance by modulating Treg metabolism and mobilization.

## 4. Effect of KLF10 in Tissue Fibrosis

The diverse functional roles of KLF10 significantly contribute to its involvement in tissue fibrosis in various organs. The regulation of TGF-β signaling, circadian rhythms, and metabolic pathways by KLF10 forms the basis for its central role in fibrotic processes.

### 4.1. KLF10 in Hepatic Fibrosis

Hepatic fibrosis is a consequence of chronic liver injury caused by various factors such as viral hepatitis, alcohol abuse, non-alcoholic fatty liver disease (NAFLD), metabolic disorders, and autoimmune diseases [59]. It is closely associated with sustained inflammation and activation of hepatic stellate cells (HSCs), the major cellular mediators of liver fibrosis. NAFLD is characterized by fat accumulation in the liver, which causes chronic inflammation and liver damage [60]. It encompasses a spectrum of liver conditions, ranging from simple hepatic steatosis (fatty liver) to non-alcoholic steatohepatitis (NASH), and in some cases, progression to advanced liver fibrosis and cirrhosis. NAFLD progression to NASH and subsequent hepatic fibrosis represents a continuum of disease severity. Several lines of evidence suggest that KLF10 is involved in hepatic diseases.

Kim et al. first examined the role of KLF10 in NASH progression [61] and found that NASH progression in mice fed a high-fat, sucrose diet significantly increased with *klf10* expression, as well as increased TGF-β and collagen genes expression in the liver. KLF10 upregulation correlated with NASH severity and the degree of liver inflammation and fibrosis, suggesting its involvement in NASH progression. Fat diet-induced NAFLD models using hepatocyte-specific KLF10 KO mice display severe NAFLD because of triglyceride accumulation and steatosis in the liver [39]. AMPK phosphorylates the KLF10 protein at Thr189 to activate and stabilize it, thereby suppressing *srebp*-*1c* expression and regulating lipogenesis and metabolic disorders. Similarly, AMPK activation in the liver reduces lipogenesis in vivo and protects against high-fructose diet-induced hepatic steatosis [62], indicating that AMPK–KLF10 axis activation could be a beneficial target for liver fibrosis treatment.

The circadian system is an internal biological clock that regulates various physiological processes in the body, including the sleep–wake cycle, hormone production, and metabolism. Many biological functions are regulated by the circadian clock, and reciprocally, metabolic interruptions can alter the rhythmic activity of metabolic pathways. Circadian rhythm disruption is involved in several diseases, including tissue fibrosis [63]. Recent studies have shown nutritional challenges in reprogramming circadian physiology and increased susceptibility to metabolic diseases in KLF10 KO mice. Leclère et al. investigated the involvement of KLF10 in liver pathology in a diet-induced steatohepatitis model [64]. They used mice fed a methionine- and choline-deficient (MCD) diet, which induced liver injury and inflammation resembling NASH in humans. The MCD diet disrupted the normal diurnal rhythm of liver biomarkers including inflammatory- and fibrosis-related genes. KLF10-deficient mice subjected to the MCD diet exhibited exacerbated liver injury, enhanced inflammation, and increased fibrosis compared to wild-type mice. These findings suggest that KLF10 may protect against steatohepatitis development by regulating the circadian regulation of associated biomarkers, and its deficiency aggravates liver injury.

KLF10 is a circadian regulator that plays important roles in liver injury and fibrosis. Guillaumond et al. and Hirota et al. have demonstrated that KLF10 expression in the liver followed a circadian rhythmic pattern, with high levels during the inactive period [51,65]. KLF10 also regulates the expression of glucose and lipid metabolism-related genes, highlighting its role in the coordination of metabolic processes with the circadian clock [52]. In addition, KLF10 expression is significantly reduced in clock-deficient Bmal1 KO mice, suggesting that Bmal1 is an upstream positive regulator of KLF10 during liver fibrosis.

Bmal1 is a master transcriptional regulator of the circadian clock that regulates biological rhythms [66] and is involved in various metabolic, inflammatory, and fibrotic diseases. Zhang et al. demonstrated that the liver-specific Bmal1 deletion caused severe liver injury and steatosis after chronic ethanol feeding [67]. In addition, a recent study has shown that Bmal1 is downregulated in the fibrotic liver tissues of mice and primary HSCs [68]. Bmal1 inhibits glycolysis in activated HSCs through regulating the isocitrate dehydrogenase 1/α-ketoglutarate (IDH1/α-KG) pathway.

**Figure 6:**
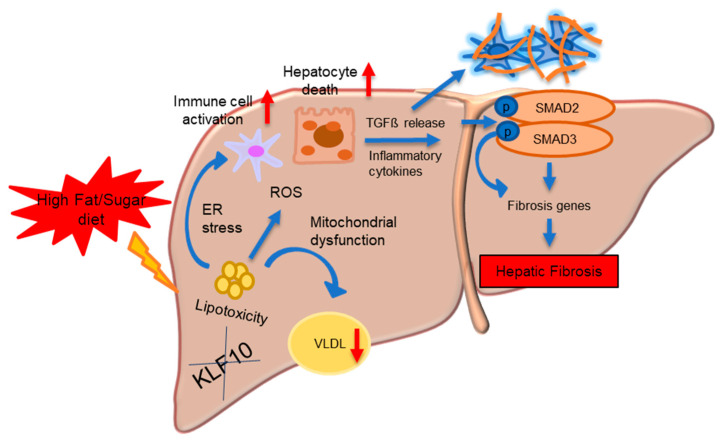
Schematic illustration of liver fibrosis in KLF10 KO mice. KLF10 is essential for TGF-β/SMAD signaling in mediating liver fibrosis. A high-fat and sucrose diet induced lipotoxicity via decreased VLDL secretion that causes reactive oxygen species (ROS) generation, ER stress, and mitochondrial dysfunction in the liver, which causes immune cell activation and hepatocyte death. TGF-β and inflammatory cytokines contribute to TGF-β-induced liver fibrosis.

Our recent studies further elucidated the role of KLF10 in hepatic health following a high-sucrose diet (Figure 6) [18,21]. Triglyceride and cholesterol concentrations as well as plasma ALT and AST levels were elevated in the KLF10 KO mice liver, whereas WT mice had minor hepatic steatosis but no apparent liver damage. Furthermore, reduced VLDL secretion may encourage lipid accumulation in the KLF10 KO mice liver. Hepatic fibrosis was observed in sucrose diet-fed KLF10 KO mice due to ER stress, inflammation, and TGF-β-dependent SMAD3 signaling activation. The most significant finding was that KLF10 deficiency increased fibrosis and collagen deposition in the mouse liver, causing hepatocyte death. In addition, KLF10 deletion activates HSCs, thus aggravating liver fibrosis [18]. Recent studies have identified that KLF10 suppresses TGF-β-induced HSC activation by targeting activating transcription factor 3 (*ATF3*) expression [21].

These results suggest that KLF10 acts as an anti-fibrotic regulator in the context of liver fibrosis. The role of KLF10 in liver injury and fibrosis may be linked to its role in circadian rhythms, ER stress, and TGF-β signaling regulation. Further studies should elucidate the mechanisms underlying the action of KLF10 in liver fibrosis.

### 4.2. KLF10 in Cardiac Fibrosis

Cardiac fibrosis is characterized by excess ECM component deposition, primarily collagen, within the myocardium. This is a common feature of various cardiovascular diseases, including hypertrophic cardiomyopathy, ischemic heart disease, and heart failure. The TGF-β signaling pathway is involved in cardiomyocyte proliferation and fibrosis.

**Figure 7:**
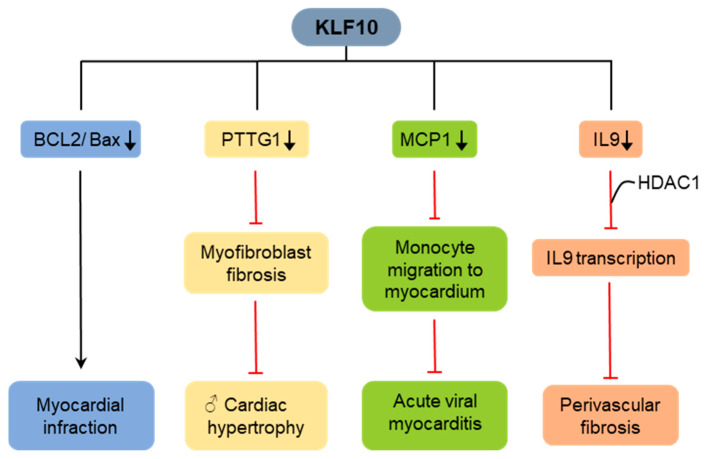
Role of KLF10 in cardiac fibrosis. KLF10 protects against cardiac fibrosis through the suppression of PTTG1, MCP1, and IL-9. KLF10 promotes cardiac fibrosis through BCL2/Bax signaling pathway.

Rajamannan et al. first investigated the effects of KLF10 deficiency on cardiac hypertrophy, which is closely associated with cardiac fibrosis [69]. KLF10 is expressed in the normal human myocardium. KLF10 KO male mice exhibited cardiac hypertrophy symptoms, such as elevated heart/body weight ratio, wall thickness, and ventricular size, compared to the control group. KLF10 is a hypertrophy suppressor that binds to the *Pttg1* promoter as one of its target genes and plays a key role in cardiac hypertrophy expansion. Gene array analysis of cardiac tissue from the left ventricles of old KLF10 KO male mice showed significantly upregulated *Pttg1* and myofibroblast fibrosis and myocyte disarray development (Figure 7) [69]. In contrast, the female mice did not develop hypertrophy or fibrosis.

Cen et al. have reported that cardiomyocytes isolated from KLF10 KO mice showed enhanced proliferation and reduced apoptosis [70]. KLF10 KO mice with myocardial infarction showed better cardiac function and smaller scar areas than C57BL/6J mice. KLF10 deficiency in myocytes and endothelial cells reduced PTEN/Akt expression and elevated Bcl-2/Bax signaling pathway levels, indicating a cardioprotective role of KLF10 (Figure 7). Although KLF10 deficiency was initially reported to be involved in cardiac hypertrophy in 2007 [69], they identified a functional role for KLF10 in myocardial infarction. Therefore, KLF10 KO provides a novel strategy for alleviating heart infarction.

KLF10 protects against virus-induced acute viral myocarditis (VMC), particularly coxsackievirus B3 (CVB3) [71]. KLF10 interacts with the MCP-1 promoter and inhibits monocyte and macrophage migration into the myocardium (Figure 7). In VMC, KLF10 levels are significantly inhibited after CVB3 infection, leading to mononuclear cell infiltration into the myocardium and VMC progression aggravation. This study elucidates the possible role of KLF10 in VMC pathophysiology and provides a new therapeutic concept for VMC immunotherapy.

KLF10 expression is elevated in hypertensive human peripheral blood mononuclear cells (PBMCs) and Ang II-treated mouse CD4+ T-cells [72]. In response to Ang II therapy, KLF10 binds to the interleukin-9 (IL-9) promoter and interacts with HDAC1 to prevent IL-9 transcription. Mice with CD4+ T-cell-specific KLF10 KO, exhibiting elevated IL-9 levels, demonstrate enhanced fibroblast intracellular calcium mobilization, activation, and differentiation. This is accompanied by an increase in collagen and ECM production, leading to the progression of perivascular fibrosis and impaired function in target organ (Figure 7). These findings suggest that the KLF10–IL-9 axis in CD4+ T-cells firmly regulates Ang II-induced perivascular fibrosis development and organ failure, potentially providing new treatment options for hypertension-related diseases.

### 4.3. KLF10 in Renal Fibrosis

Renal fibrosis is a common manifestation of various chronic kidney diseases including diabetic nephropathy, glomerulonephritis, and interstitial nephritis. It is characterized by tubulointerstitial fibroblast proliferation, excess ECM deposition, and inflammatory cell infiltration. Fibrosis disrupts the normal kidney structure, leading to progressive renal function loss and, eventually, kidney failure.

The TGF-β/SMAD signaling pathway is involved in renal fibrosis development and progression. SMAD7 is a negative feedback regulator of TGF-β/SMAD signaling pathways and KLF10 represses *SMAD7* transcription. Wahab et al. investigated TGF-β/SMAD signaling modulation in mesangial cells by CTGF, a profibrotic factor involved in fibrosis in multiple tissues [73]. CTGF induces KLF10 and concurrently enhances TGF-β/SMAD signaling via transcriptional repression of *SMAD7*. SMAD7 expression decreased in various fibrotic diseases including renal fibrosis [74]. Although the specific role of KLF10 in renal fibrosis was not explored in this study, their findings suggest its potential involvement in TGF-β signaling modulation and renal fibrosis development.

**Figure 8:**
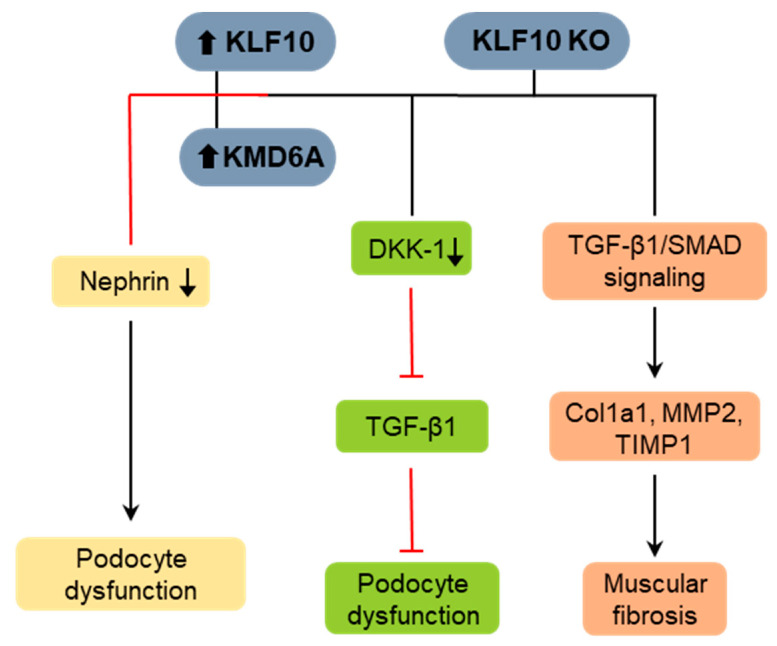
Role of KLF10 in renal and muscle fibrosis. KLF10 knockout (KO) ameliorates diabetic renal fibrosis by the downregulation of DKK-1 expression and inhibition of TGF-β1. Under diabetic conditions, podocytes exhibit increased expression of KLF10 and KMD6A, leading to podocyte dysfunction through the inhibition of nephrin. In dystrophic skeletal muscles, KLF10 KO leads to increased expression of collagen and fibronectin genes, a process that is enhanced by TGF-β1/SMAD signaling.

Diabetes-induced renal fibrosis (DN), the most frequent complication of type 2 diabetes, is a major cause of end-stage renal diseases worldwide [75]. A recent study has demonstrated the involvement of KLF10 in renal fibrosis, specifically diabetic nephropathy [75]. Various fibrosis markers, such as TGF-β, collagen, and fibronectin, were significantly downregulated in KLF10 KO mice compared to those in control diabetic mice. KLF10 depletion improved diabetes-induced renal fibrosis by downregulating Dickkopf-1 (DKK-1) expression, which was associated with reduced Wnt/β-catenin signaling activity (Figure 8). DKK-1 induction by β-amyloid (Aβ) significantly activates KLF10 expression in primary neuronal cultured cells and causes Alzheimer disease [76]. These results suggest that KLF10 could be a potential therapeutic target for diabetes-induced renal fibrosis.

KLF10 also modulates crucial physiological functions in the kidney, including glomerular endothelial cell maintenance and podocyte function [77]. Podocytes are specialized kidney cells that play a vital role in maintaining the renal glomerular filtration barrier. Podocyte dysfunction is a characteristic feature of diabetic kidney disease. KLF10 expression was positively regulated by histone demethylase lysine demethylase 6A (KDM6A) in podocytes under diabetic condition (Figure 8) [78]. In addition, KLF10 directly binds to the promoter region of the nephrin gene, specifically an SP1-binding site, and subsequently recruits the methyltransferase Dnmt1. Through this interaction, KLF10 acts as a transcriptional repressor of the nephrin promoter. Nephrin is a genetic marker of podocytes, and its absence causes kidney dysfunction. Importantly, both KLF10 and KDM6A mRNA and protein levels increased in kidney tissues of patients with diabetic nephropathy. Therefore, KLF10 plays a distinct role in promoting DN, making it a promising therapeutic target for diagnosing diabetic nephropathy.

### 4.4. KLF10 in Muscular Fibrosis

Fibrosis can occur in other organs, such as the skeletal muscle and skin. Skeletal muscle fibrosis is a defining feature of muscular dystrophies where large myofiber areas are replaced by the progressive deposition of collagens and other ECM proteins produced by muscle fibroblasts. KLF10 is abundant in the skeletal muscles and plays a vital role in regulating skeletal muscle function [19]. DiMario et al. compared the effects of KLF10 depletion in wild-type and X-linked muscular dystrophy mice [19]. KLF10 depletion in dystrophic skeletal muscles increased collagen and fibronectin gene expression, thus enhancing fibrosis (Figure 8). The grip strength of KLF10 KO dystrophic mice also decreased. Further, Smad2 was scarcely detectable in both wild-type and X-linked muscular dystrophy mice diaphragms, suggesting that KLF10 may suppress *Smad2* expression in the diaphragm. The findings of this study indicate that KLF10 plays a crucial role in mediating the fibrotic effects of TGF-β signaling in chronically damaged regenerative muscles. Recently, KLF10 has been identified as a key regulator of the contractile behavior of skeletal muscle fibers, exhibiting muscle fiber-type-specific functions that contribute to muscle homeostasis [79]. These findings not only emphasize the significance of KLF10 in skeletal muscle function but also provide valuable insights into the molecular mechanisms governing the contractility of skeletal muscle fibers.

### 4.5. KLF10 in Pulmonary Fibrosis

Pulmonary fibrosis (PF) is a chronic and progressive disease characterized by irreversible scarring and remodeling of the lung tissue. Idiopathic pulmonary fibrosis (IPF), the most common type of PF, presents particular challenges due to its unknown etiology and lack of curative treatment. Although the pathophysiology of PF has not yet been well clarified, it is believed to involve the activation of inflammatory cells by extrinsic irritants, fibroblast recruitment, and a sustained fibrotic response [80].

The role of KLF10 in suppressing TGF-β-induced EMT was reported in lung cancer, demonstrating that KLF10 functions as a transcriptional repressor, particularly of the EMT-promoting transcription factor SLUG/SNAI2, thereby limiting the ability of TGF-β to induce EMT [81]. This finding is particularly relevant to PF, as EMT plays a central role in the disease’s progression.

While KLF10’s role in fibrosis in various tissues is established, its specific role in PF remains less defined. However, Huang LT et al.’s investigation into KLF10 in regulating the inflammatory response in bronchoalveolar lavage (BAL) fluid immune cells and lung tissue highlights its significance in chronic pulmonary disease pathogenesis [82]. KLF10 deficiency in mice exacerbates pulmonary inflammation, as evidenced by increased expression of the pro-inflammatory molecule Natriuretic Peptide Receptor Type A (NPRA), indicating KLF10’s critical role as a transcriptional repressor of NPRA. KLF10 KO mice exhibited heightened sensitivity to lipopolysaccharide or ovalbumin challenge, showing severe pulmonary neutrophil accumulation and inflammatory changes in the lungs.

These findings suggest NPRA as a potential KLF10-regulated target in PF. Prior research has shown that NPRA knockout can prevent lung inflammation in allergic asthma models, indicating a similar therapeutic potential in PF. Targeting the KLF10-NPRA pathway could offer new treatment avenues for pulmonary inflammation and potentially fibrosis in PF and IPF.

In summary, while the specific role of KLF10 in PF and IPF is yet to be fully defined, emerging research points to its significant impact on pulmonary inflammation and potential fibrotic processes. Understanding KLF10’s role in these pathways opens up new possibilities for therapeutic interventions in PF, particularly in modulating inflammatory responses that contribute to fibrotic progression.

### 4.6. KLF10 in Skin Fibrosis

Keloids, a skin fibrosis-associated disorder, are characterized by an abnormal wound healing process that deposits excess ECM components in the dermis. The TGF-β pathway plays a crucial role in keloid pathogenesis, contributing to fibroblast proliferation and collagen accumulation in these fibrotic lesions. SMAD7 is downregulated in keloid scars and fibroblasts [83,84]. Hu et al. demonstrated that KLF10 expression increased significantly in keloid fibroblasts, while SMAD7 levels decreased [41]. KLF10 knockdown decreased collagen content, proliferation, and migration of keloid fibroblasts. These findings offer valuable insights into the role of KLF10 in mediating TGF-β signaling and its implications in keloid scar development.

## 5. Conclusions and Perspectives

KLF10, a member of the KLF family of transcription factors, plays a crucial role in tissue homeostasis and fibrosis, a condition marked by excessive ECM accumulation and fibrous tissue formation. It regulates genes involved in ECM production, such as collagen and fibronectin, and balances ECM production and degradation, impacting tissue remodeling and repair. KLF10 also affects fibroblast-to-myofibroblast differentiation and HSC activation, and regulates immune responses and inflammation, which are key in fibrosis.

Its role in fibrosis is complex and varies by tissue. KLF10 acts as a positive regulator in TGF-β/SMAD signaling, contributing to renal and skin fibrosis, while protecting against cardiac and skeletal muscle fibrosis. Its deficiency exacerbates liver injury and fibrosis, highlighting its potential anti-fibrotic effects through mechanisms like ER stress, TGF-β signaling, and circadian rhythm regulation.

Understanding the tissue-specific regulation and molecular mechanisms of KLF10 in fibrosis, as well as its interactions with other regulators and pathways, is essential for comprehensive insights. Its clinical relevance in fibrotic diseases could make KLF10 a potential diagnostic marker or therapeutic target. Dysregulated KLF10 expression in various fibrotic conditions, correlating with severity or improvement, suggests its diagnostic potential. Measuring KLF10 expression could aid in fibrosis diagnosis and monitoring, though more research is needed to confirm its diagnostic value and clinical utility.

Targeting KLF10 in therapeutic strategies seems promising, given its involvement in fibrogenesis. Modulating KLF10 or its downstream pathways could influence the fibrotic process. Since KLF10 is integral to various signaling pathways, targeting these pathways might affect KLF10 activity, offering anti-fibrotic strategies. In summary, KLF10 is a key emerging player in tissue fibrosis. Understanding its roles and dysregulation is vital to develop diagnostic and therapeutic strategies. Continued research on its mechanisms, diagnostic value, and therapeutic potential will enhance fibrosis research and potentially improve clinical outcomes in fibrotic diseases. The potential of KLF10 as an early biomarker, supported by current research and non-invasive diagnostic techniques, is particularly promising in cases of liver and lung fibrosis.

## Data Availability

Data is contained within the article.

## Abbreviations

AMPK	AMP-activated protein kinase
CDK2	Cyclin-dependent kinase 2
ChREBP	Carbohydrate response element-binding protein
ECM	Extracellular matrix
HSC	Hepatic stellate cell
KLF10	Krüppel-like factor 10
KO	Knockout
NAFLD	Non-alcoholic fatty liver disease
NASH	Non-alcoholic steatohepatitis
SIAH1	Seven in Absentia homologue-1
SMAD	Suppressor of mothers against decapentaplegic
TGF-β	Transforming growth factor-beta
VMC	Viral myocarditis
